# Sex influence on muscle synergies in a ballistic force-velocity test during the delayed recovery phase after a graded endurance run

**DOI:** 10.1016/j.heliyon.2022.e09573

**Published:** 2022-06-09

**Authors:** Robin Macchi, Alessandro Santuz, Arnaud Hays, Fabrice Vercruyssen, Adamantios Arampatzis, Avner Bar-Hen, Caroline Nicol

**Affiliations:** a ISM, CNRS & Aix Marseille University, Marseille, France; b Department of Training and Movement Sciences, Humboldt-Universität zu Berlin, 10115 Berlin, Germany; c Berlin School of Movement Science, Humboldt-Universität zu Berlin, 10115 Berlin, Germany; d IAPS, University of Toulon, Toulon, France; e CEDRIC, Conservatoire National des Arts et Métiers, Paris, France

**Keywords:** Fatigue, Women, Endurance running, EMG, Explosive movement, Jump, Non-negative matrix factorization

## Abstract

The acute and delayed phases of the functional recovery pattern after running exercise have been studied mainly in men. However, it seems that women are less fatigable and/or recover faster than men, at least when tested in isometric condition. After a 20 km graded running race, the influence of sex on the delayed phase of recovery at 2–4 days was studied using a horizontal ballistic force-velocity test. Nine female and height male recreational runners performed maximal concentric push-offs at four load levels a week before the race (PRE), 2 and 4 days (D2 and D4) later. Ground reaction forces and surface electromyographic (EMG) activity from 8 major lower limb muscles were recorded. For each session, the mechanical force-velocity-power profile (i.e. theoretical maximal values of force ($\bar{\text{F}}$ 0), velocity ($\bar{\text{V}}$ 0), and power ($\bar{\text{P}}$ max)) was computed. Mean EMG activity of each recorded muscle and muscle synergies (three for both men and women) were extracted. Independently of the testing sessions, men and women differed regarding the solicitation of the bi-articular thigh muscles (medial hamstring muscles and rectus femoris). At mid-push-off, female made use of more evenly distributed lower limb muscle activities than men. No fatigue effect was found for both sexes when looking at the mean ground reaction forces. However, the force-velocity profile varied by sex throughout the recovery: only men showed a decrease of both $\bar{\text{V}}$ 0 (p < 0.05) and $\bar{\text{P}}$ max (p < 0.01) at D2 compared to PRE. Vastus medialis activity was reduced for both men and women up to D4, but only male synergies were impacted at D2: the center of activity of the first and second synergies was reached later. This study suggests that women could recover earlier in a dynamic multi-joint task and that sex-specific organization of muscle synergies may have contributed to their different recovery times after such a race.

## Highlights

• The use of bi-articular thigh muscles differed between men and women.
• Muscle synergies were sex dependent.
• Only men showed decreased theoretical maximal velocity and power 2 days after running.
• Centers of activity of muscle synergies were shifted later for men 2 days after the race.
• Sex-specific organization of muscle synergies may explain the different recovery times.

## 1 Introduction

With the growing involvement and success of women in endurance running races, studies have been conducted during the last three decades on the biomechanical and physiological characteristics of women compared to men during exercise [1, 2]. Across a range of speeds [1, 3, 4, 5] and surface inclines [3], female recreational runners are characterized by specific kinematic movements and neuromuscular mechanisms [6] compared to male runners. For instance, in terms of neuromuscular control, female runners are reported to have a higher gluteus maximus activity during the stride cycle, regardless of speed (1.8–3.6 m/s) and gradient (0–15% grade) [3, 7]. Looking at the specific phases of running, women present a higher preactivation of the peroneal muscle group [8] than men but, conversely, a lower activation of this muscle group during the braking and push-off phases. Interestingly, sex differences are influenced by the gradient as it has been reported for female runners, a higher preactivation of the medial hamstring muscles during a downhill treadmill run (−15° at 7.5 km/h) [9] and a greater increase in activation of the vastus lateralis with increasing inclination [3]. Based on the differences in muscle activation reported between male and female runners, it can be expected that a strenuous running task would result in sex-selective muscle fatigue.

After exhaustive stretch-shortening cycle (SSC) type exercises such as endurance running, the functional recovery pattern has been studied mainly in men and reported to be testing-time dependent and biphasic in nature [10]. Acute functional decreases, mainly attributed to metabolic fatigue, partially recover within 2 h, while secondary central and reflex inhibition and functional decreases, related to the inflammatory/remodeling process after ultrastructural muscle damage, usually peak one to two days later and last for 8–15 days. The secondary recovery phase may be associated with delayed onset muscle soreness (DOMS) resulting from sensitization of the nociceptive part of muscle afferents III and IV around the inflammatory peak. DOMS thus appear late but also disappear early despite the subsistence of protective and/or compensatory neural adjustments that could represent a potential source of injury when people consider that they have recovered. Compared to men, women are generally characterized by smaller muscle mass, greater muscle perfusion and a relatively greater surface area of type I muscle fibers, resulting in less metabolite production, sensitization of muscle afferents, and muscle inhibition that would potentially result in less fatigue than in men [11, 12, 13]. Given the early disappearance of DOMS and the usual tendency of runners to train as soon as the pain disappears, it seems necessary to characterize their exact delayed recovery pattern in order to define the optimal time to resume running and thus to prevent injury.

Surprisingly, only a few studies have investigated the endurance running-induced fatigue effects in women [14]. Compared to male runners in the acute recovery phase, females showed attenuated peripheral plantar flexor fatigue and a smaller decrease in maximal voluntary contraction (MVC) of knee extensors after ultra-endurance races [15, 16]. A smaller acute decrease in knee extensor MVC was also reported after a 20 km level run [17, 18], while Boccia et al. (2018) [19] found no sex difference. Recently, our research group investigated the functional recovery pattern up to 4 days after a graded 20 km race [18] and found an earlier recovery in female runners but this was dependent on the testing task. Indeed, at 4 days, only men showed decreases in bilateral knee extension MVC and maximal power in drop jump, while similar recovery time was found in unilateral knee extension MVC tests and velocity at take-off in drop jump. In the pure concentric tests, no fatigue effects were observed in either men or women. Thus, women might be less prone to damage after endurance running race, potentially due to the protective effects of sex hormones, particularly estrogens which are antioxidant, membrane stabilizer and satellite cell proliferators [20]. On the other hand, while DOMS was reported for the quadriceps independently of sex 2 days after the race, only female runners reported then DOMS for the hamstring muscle group. The observed dependence of sex differences on task and muscle underlines the need to further evaluate the associated neural adjustments.

Neuromuscular adjustments to SSC-type fatigue have been studied mainly in men and shown to be testing-task dependent [e.g. 21], with multi-joint maximal dynamic tests being considered more mechanistically meaningful than conventional MVC tests [10, 22, 23]. Unlike maximal SSC testing tasks with ground impacts (e.g. drop-jump), maximal dynamic tests involving slight or no impact (counter-movement or squat jumps) are usually showing no significant decreases in performance, or even improvements attributed to a change in neural strategies [24]. In this context, García-Ramos et al. (2018) [25] recently demonstrated the interest of the force-velocity relationship (FV) to assess the effects of fatigue on the distinct abilities of muscles to produce force, velocity, and power output while performing a multi-joint maximal ballistic task. The linear force-velocity and polynomial power-velocity relationships depend on individual muscle-tendon structural and mechanical properties as well as on the neural activation [26]. These relationships can be summarized by three typical parameters: the theoretical values of the maximal force at zero velocity ($\bar{\text{F}}$ 0) and maximal velocity at zero force ($\bar{\text{V}}$ 0), and the maximal power output ($\bar{\text{P}}$ max). García-Ramos et al. (2018) [25] found, for ballistic upper limb movements, that the model remained linear after various fatigue protocols while revealing potentially specific decreases in force or velocity. Yet, to the best of our knowledge, the force-velocity mechanical profile in ballistic movement has never been assessed for the lower limbs after an endurance running task. In addition, the running task has been reported to be asymmetric ranging from 3 to 54% when looking at kinetic variables [27]. Therefore, it is of particular interest to distinguish the dominant from the non-dominant limb for the force-velocity test in a bilateral push-off. Although to our knowledge there is no much scientific knowledge on the effect of unilateral fatigue on bilateral tasks, Marchetti et al. (2011) [28] showed an increase in the asymmetry index in a bilateral jump after a unilateral fatigue task.

Considering the protective *vs*. compensatory neural adjustments and the different neural strategies reported to take place depending on the testing time and task after an exhaustive endurance run [10], it seems of particular interest to perform electromyographic (EMG) recordings of the main lower limb muscles during a FV test. Complementary EMG analyses may then be used to explore the richness of the neuromuscular adjustments to fatigue in relation with sex-differences. The root mean square (RMS) is considered as the main method in the time domain [29]. Considering that movements are not the result of independent muscle activations, but rather of linear combined patterns of activations (called muscle synergies) [30], complex mathematical models, including linear machine learning such as non-negative matrix factorization (NMF), seem well-suited to assess such a dynamic task. This method has been used to quantify muscle synergies in a wide variety of tasks [31, 32, 33, 34] and seems particularly suitable for revealing potential sex-differences in muscle fatigue and intermuscular compensations during the recovery period.

To this end, this study focused on the sex influence on the horizontal force-velocity (FV) profile and associated muscle activation pattern of major lower limb muscles before as well as 2 and 4 days after a 20 km graded running race. The hypotheses were that (i) the FV profile would change less in women than in men during the delayed recovery period, and (ii) the muscle activity pattern would be sex-dependent before fatigue and evolve differently during the 4 days of recovery. This study aims to improve the knowledge of delayed neuromuscular fatigue in women through the analysis of movement coordination in order to determine their optimal time to resume running and thus prevent injuries.

## 2 Method

### 2.1 Participants

All participants were recreational runners who had registered 6 months earlier to take part to the international Marseille-Cassis race of 20 km including positive and negative gradients (+382 m and −294 m). Prior to the experiment, the sample size was calculated using G Power (Version 3.1.9.7). According to the experimental design and for a medium effect size (i.e. η2 = 0.06), 14 participants in each sex group were required to obtain a statistical power of 80%. Due to the loss of three participants (two because of back pain and torn muscle during the race and one for noisy EMG recordings), the final group included only 9 female (age: 35 ± 7 years (26–45), body mass: 59.8 ± 8.7 kg, height: 1.66 ± 0.08 m) and 8 male (age: 29 ± 7 years (21–38), body mass: 70.9 ± 6.2 kg, height: 1.76 ± 0.06 m) runners. Two women were in the follicular phase, 4 in the luteal phase and 3 were amenorrhoeic. The current study was part of a larger study on the effects of graded running race on fatigue [18] which was approved by the local ethics committee (2019-15-09-35) and, in accordance with the Helsinki Convention, written informed consent was obtained from all runners.

### 2.2 Experimental design

**Figure 1:**
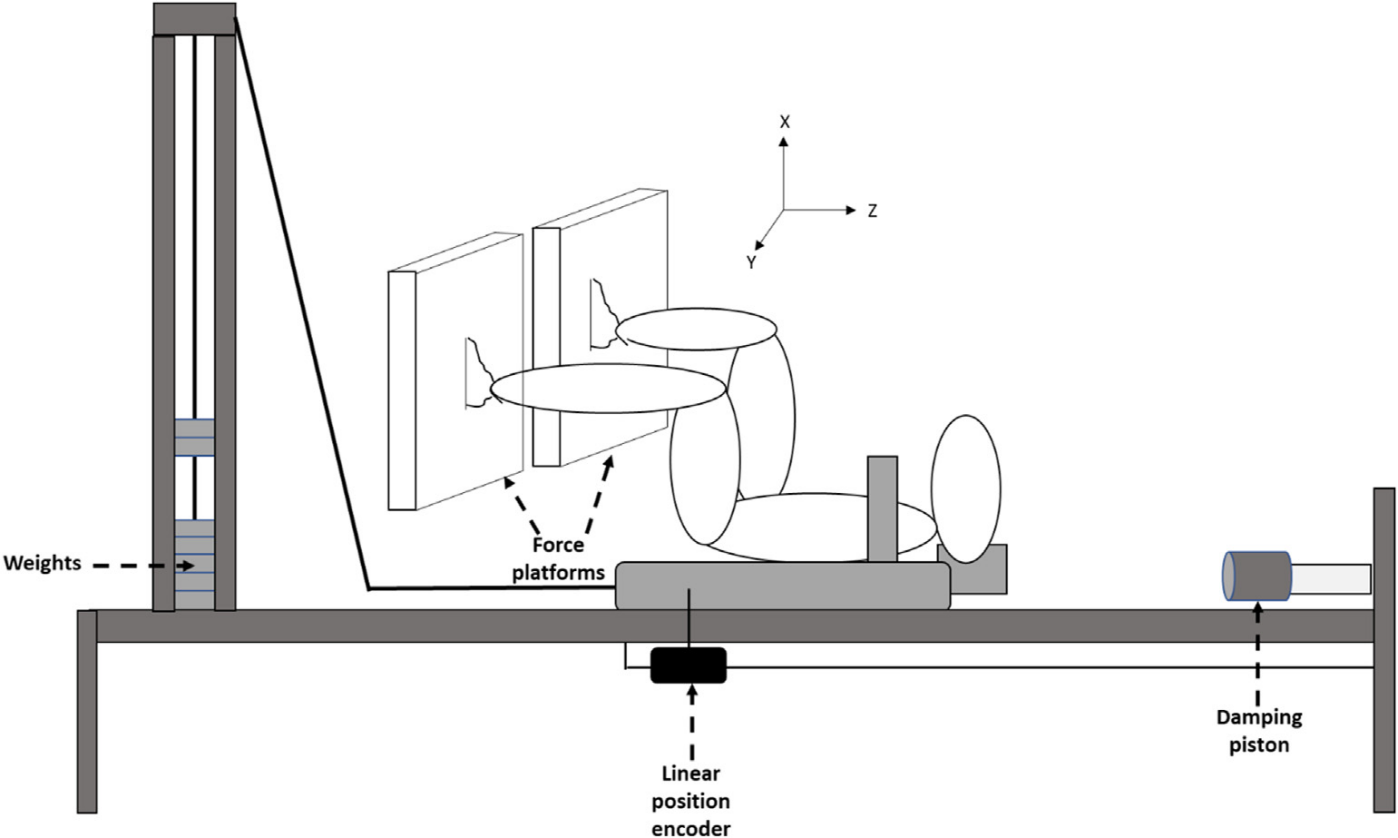
Experimental set-up on the horizontal force-velocity ergometer.

The experimental design included a familiarization and three experimental sessions: a week before the running race (PRE), 2 and 4 days later (D2 and D4, respectively). As far as possible, the 3 test sessions were individually scheduled at the same time of day. Each experimental session started by checking the runner's body mass on a Tanita scale (MC980MA Tanita) before performing a sex-standardized 10 min warm-up. Uni- and bilateral maximal isometric voluntary contractions of the knee extensors (MVC), a squat jump (SJ), and a drop jump (DJ) tests as well as delayed muscle soreness (DOMS) for the quadriceps, hamstring and triceps surae muscle groups were evaluated and reported previously [18]. The participants were then equipped with surface EMG electrodes before performing a FV test in the supine position. This ergometer (Figure 1) allows the assessment of participants' peak performance while minimizing the risk of injury, and providing a good standardized position as it is known to impact on the results [35].

### 2.3 Measurement protocol

The horizontal FV (HFV) test consisted of two trials of ballistic squat jumps performed in a supine position on a frictionless sled (INPI deposit n°: FR2011204) at 4 load levels: 0, 20, 40 and 60% bodyweight (BW) (Figure 1). The dynamic conditions were performed in a random order among the participants. For a given runner, the order of the testing conditions remained the same in each session. The starting position was held still for 2–3 s with hip, knee and ankle joints at 90°. They were asked to apply their force as rapidly as possible on the force plates (one under each foot) in order to reach the highest velocity at take-off. All trials were performed on two Kistler force plates (Kistler 9260AA3 (0.5 × 0.3 m)) which recorded the three-dimensional (3D) ground reaction force (GRF) components produced by the dominant and non-dominant lower limbs. Horizontal displacement over time was recorded with an accuracy of 0.1 cm using a linear encoder attached to the sled.

Surface EMG activity was recorded from 8 muscles of the dominant lower limb using DTS EMG sensor Model 542 (Noraxon Inc., USA): *vastus medialis*, *vastus lateralis*, *rectus femoris*, *medial hamstrings*, *tibialis anterior*, *gastrocnemius medialis*, *gastrocnemius lateralis* and *soleus*. To keep skin impedance low, the site for electrode placement was prepared by shaving, gently abrading the skin using sandpaper and cleaning with 70% isopropyl alcohol. Bipolar electrodes were placed in accordance with Seniam recommendations [36]. To secure an identical EMG electrode placement among sessions, their location was precisely marked on the skin using an indelible marker. All signals were recorded continuously at 1.5 kHz and synchronized by the means of an external trigger. To define the dominant lower-limb, the runners had to climb up and down a 50 cm high wooden box with their self-selected lower limb designed as the dominant limb.

### 2.4 Data processing

**Figure 2:**
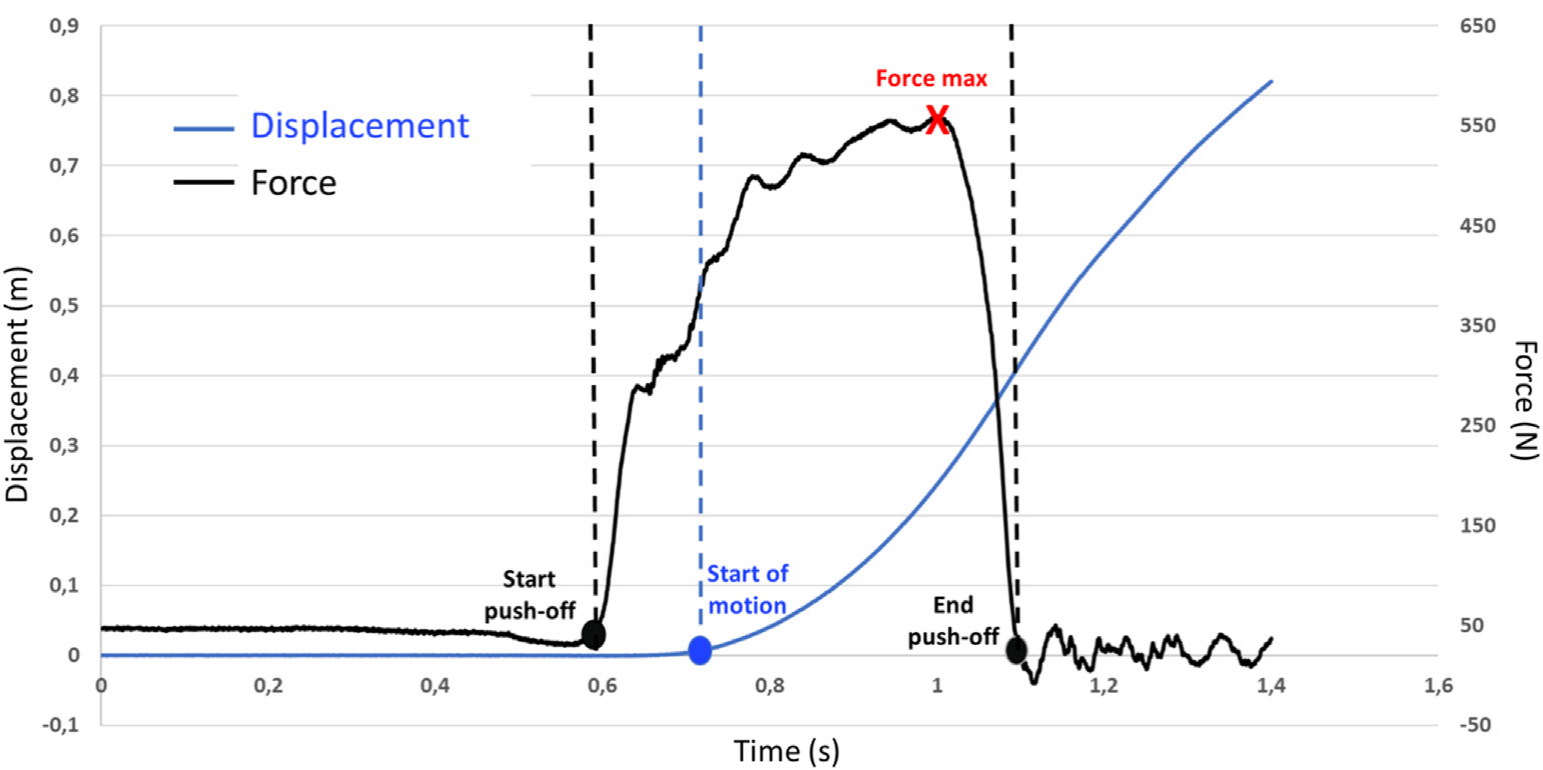
Horizontal force of the dominant limb (in black) and displacement (in blue) curves as a function of time for a typical participant during the 40% BW resistive condition. The two vertical dotted black lines represent the beginning and the end of the push-off. The dotted blue line represents the beginning of the first measurable displacement named “start of motion”.

Data processing was performed using custom Matlab (Matworks Inc, Novi, USA) and R (R v3.6.3, R Core Team, 2020, R Foundation for Statistical Computing, Vienna, Austria) routines. The push-off start was defined as the inflexion point of the force curve and the end of push-off was set when the normal component of the ground reaction force fell to zero (Figure 2). Since the first phase of the push is motionless (Figure 2) due to the high inertia of the device, for the force-velocity variables, the onset of the average force was set as the first measurable displacement in order to calculate the average force and velocity at a similar time. As the push-off distance may influence the results, it was computed and checked that there was no significant change between the loads and the sessions.

### 2.5 Force-velocity processing

**Figure 3:**
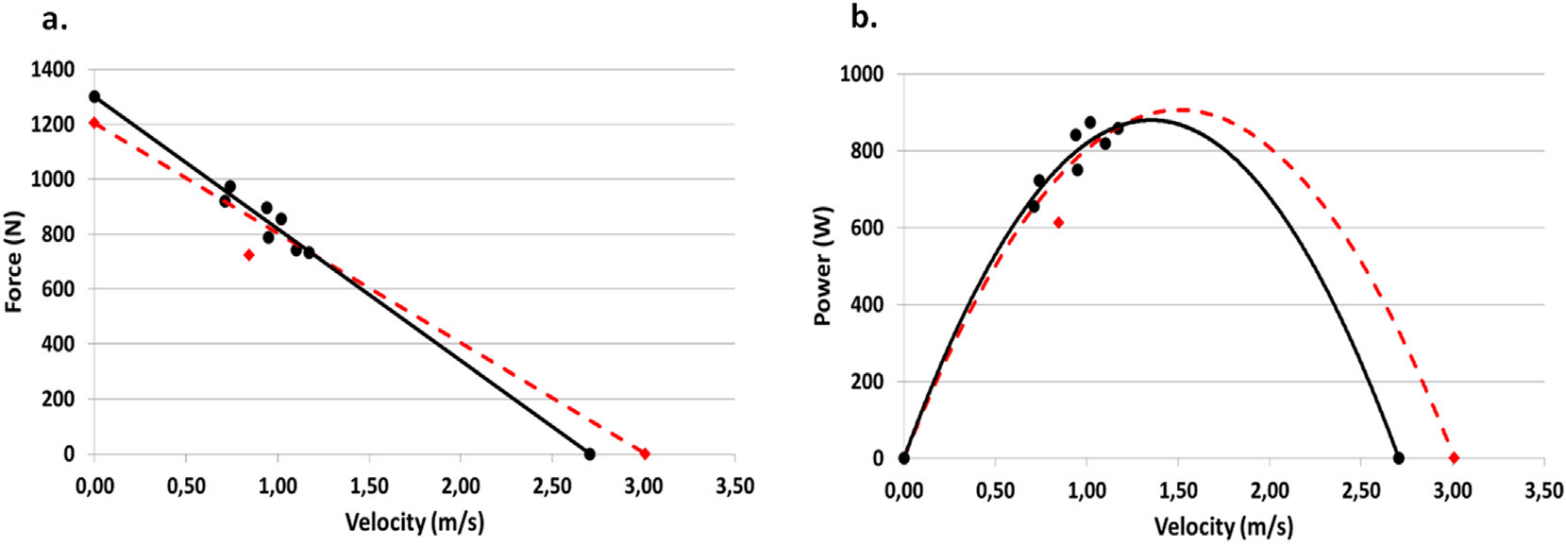
Illustration for a given female runner of the automatic selection of the trials kept for the force-velocity (a) and power-velocity (b) relationships in the PRE-session. The black points represent the selected trials whereas the red point represents the deleted trial (1/8). The straight lines and curves represent the force-velocity and power-velocity relationships. The black ones correspond to the selected trials whereas the red cut lines correspond to all trials.

Force-velocity relationships were determined by least-squares linear regressions using the average normal force component and velocity at each load. Individual force-velocity slopes were extrapolated to obtain the intercepts corresponding to the theoretical maximal values of “$\bar{\text{F}}$ 0” and “$\bar{\text{V}}$ 0” [26]. Then, the theoretical peak value of the velocity-power polynomial (second-degree) relationships was obtained and noted as “$\bar{\text{P}}$ max” [26]. The force-velocity-power relationships were fitted for both legs (FV_bilateral_) as well as for the dominant and non-dominant legs separately. A selection among the 8-dynamic push-offs was performed for each participant to obtain the most accurate linear FV relationship due to a potential hip lift, slight countermovement or non-maximal trial which could distort linearity. To perform this, an automatic selection was set from a Matlab routine. This routine deleted the points with residuals superior to 100 N, beginning by those below the linear regression. The same routine was applied on the velocity-power polynomial relationship. In addition, the routine checked that computed value of “$\bar{\text{P}}$ max” was close to the actual power achieved. Finally, the automatic selection was compared to the manual selection and the mismatch was computed. In the literature, manual selection is the most used and consists in visually selecting the right trials based on the experimenter's expertise. Figure 3 presents an example of the automatic selection for one participant.

Each polynomial and linear relationships (without the extrapolated $\bar{\text{F}}0$ and $\bar{\text{V}}0$ parameters) were assessed by the coefficient of determination (R^2^) representing the variance explained by the model. The slope of the linear FV relationship (noted S_FV_) was computed as follows [26]:

$$S_{FV}=\;-\frac{\bar{\text{F}}0}{\bar{\text{V}}0}$$

To analyze the imbalance between the dominant and non-dominant leg FV relationships, the asymmetry index (SI) was computed between the S_FV_ of the dominant and non-dominant legs as follow [37]:

$$SI_{SFV}=\left(\frac{\left|S_{FV}\left(\text{Ldom}\right)-S_{FV}\left({\text{Lnon}}_{\text{dom}}\right)\right|}{\text{avg}\left(S_{FV}\left(\text{Ldom}\right),S_{FV}\left({\text{Lnon}}_{\text{dom}}\right)\right)}\right)$$

Where Ldom and ${\text{Lnon}}_{\text{dom}}$ correspond to the dominant and non-dominant lower limbs, respectively.

The mean 3D GRF were computed from the actual onset of push-off (i.e. the breakpoint between the stabilized force and the rate of force development) to account for the force produced to overcome the inertia of the system.

To quantify the push-off effectiveness and potential compensations, the effective force ratio was computed as the mean normal force component (horizontal performance axis) divided by the mean of the resultant force in 3D [38]. The rate of force development was computed using a linear least-squares linear regression fitting all points from the first 75 ms from the onset of the push-off and then the best trial was selected for each load [39].

### 2.6 Mean muscle activity

As previously described [40], the raw EMG signals were band-pass filtered with cut-off frequencies between 10 and 500 Hz. Then, the signals were high-pass filtered (cut-off frequency 50 Hz), full-wave rectified and lastly low-pass filtered (cut-off frequency 20 Hz) to obtain a linear envelope using a 4^th^ order IIR Butterworth zero-phase filter. After subtracting the minimum, the amplitude of the EMG recordings was normalized by the maximal EMG activity recorded among the 8 trials of the PRE-session. Each push-off was time-normalized to 100 points to allow inter-participant comparisons.

The mean activity of each recorded muscle was computed from a root mean squared (RMS) analysis for the global push-off and for its first (0–25%), middle (25–75%) and last (75%–100%) parts.

### 2.7 Muscle synergies

Muscles synergies data were extracted from the recorded EMG activities (normalized by the maximal EMG activity of the session) of all trials through a custom script [41] using the classical Gaussian non-negative matrix factorization (NMF) algorithm [34, 40]. NMF is an iterative optimization method that minimizes the normative error-matrix computed as:

E=||M−V||

Where M is the *m* × *n* (m rows and n columns) matrix of the initial 8 time-dependent muscle activity vectors. V represents the new matrix reconstructed multiplying the two matrices W and H, approximates the initial matrix M. W is an *m* × *s* matrix containing the relative activities of each muscle (*i.e.* motor modules), where *s* is the number of synergies selected for extraction and H is an *s* × *n* matrix containing the time-varying activity of each synergy (*i.e.* motor primitives) [42]. The update rules for W and H are presented in Eqs. (1) and (2) (Eq1 and Eq2 respectively).

(1) $$\left\{ \begin{array}{l} H_{i+1}=\;H_{i}\frac{W_{i}^{T}V}{W_{i}^{T}W_{i}H_{i}}\\ W_{i+1}=\;W_{i}\frac{V{\left(H_{i+1}\right)}^{T}}{W_{i}H_{i+1}{\left(H_{i+1}\right)}^{T}} \end{array}\right.$$

Where “*T* “represents the transposed of the matrix.

The quality of the reconstruction was assessed by the coefficient of determination (R^2^) between the matrix M and V. For each synergy, the iterations were stopped when the change in the R^2^ was smaller than the 0.01% in the last 20 iterations [43] meaning that, with this amount of synergies, the signal could not be reconstructed any better. This operation was first completed by setting the number of synergies to one. It was then repeated by increasing the number of synergies, up to a maximum of 6 synergies, which was chosen to be equal to 75% of the number of muscles. For each synergy, the factorization (NMF) was repeated 10 times, each time creating new randomized initial matrices W and P, in order to avoid local minima [44]. The solution with the highest R^2^ was then selected for each of the 6 synergies. To choose the minimum number of synergies required to represent the original signals, the curve of R^2^ values *vs.* synergies was fitted using a simple linear regression model using all 6 synergies. The mean squared error [45] between the curve and the linear interpolation was then calculated. Afterwards, the first point in the R^2^-*vs*.-synergies curve was removed and the error between this new curve and its new linear interpolation was calculated. The operation was repeated until only two points were left on the curve or until the mean squared error fell below 10^−4^. This was done to search for the most linear part of the R^2^-*vs*.-synergies curve, assuming that in this section the reconstruction quality could not increase considerably when adding more synergies to the model.

The recognition of fundamental synergies (which can be defined as an activation pattern a single main peak of activity in the motor primitive) was performed by clustering similar motor primitives and motor modules by NMF, using the same algorithm employed for synergy extraction with the number of synergies set to the maximum factorization rank plus one. For each synergy, the center of activity (CoA) was defined as the angle of the vector (in polar coordinates) that points to the center of mass of that circular distribution [46] using the following equations:

$$\left\{ \begin{array}{l} A=\;\overset{100}{\underset{t=1}{\sum }}\left(\cos\;\theta \times \;P_{t}\right)\\ B=\;\overset{100}{\underset{t=1}{\sum }}\left(\cos\;\theta \times \;P_{t}\right) \end{array}\right.$$

$$CoA=\text{arctan}\left(\frac{B}{A}\right)$$

The full width at half maximum (FWHM) representing the duration of activation patterns [47] was also computed as the number of points exceeding half maximum of the synergy. For each condition, the number of clusters was defined as the maximum of synergies found by the NMF. If two or more fundamental synergies were blended into one, the synergy was classified as “combined”. In our dataset, combined synergies represented between 13% to 36% of the total synergies extracted. Furthermore, fundamental synergies can be compared given their similar function (i.e. motor primitives and motor modules are comparable since they serve a specific task), combined synergies often differ from one another making their classification impossible. Thus, combined synergies were excluded from the analysis although they could be interesting for an individual analysis.

### 2.8 Statistics

For each variable, a linear mixed model was performed from the *lmerTest* package [48] and *lme 4* package [49]. Restricted maximum Likelihood (REML) estimation was used in the model. Sex, session, load, trial and muscle (for EMG variables) were selected as fixed effects. The intercepts for the participants were chosen as random effect as well as the random slope by-participants for the session effect. For all models, the significance of the random factors was tested. The best model (i.e number of fixed effects and interactions) was chosen by likelihood ratio tests of model comparisons using a backward selection method. Then an ANOVA (degrees of freedom estimated with Satterthwaite formula) was performed on the selected model. This was followed by pairwise comparisons with Tukey's (for main effect) or Holm's (for interaction) adjustment. For the synergy variables, “synergy’ was added as a fixed factor and the outliers were removed from a Grubbs's test performed on the residuals.

Despite the high robustness of the mixed model [50], before each mixed model, normality was verified from a Shapiro-Wilk test on the residuals. In addition, the independence between random variables and residuals were checked from the deviance profile. If an assumption was violated, ANOVA or mixed-model by permutation with 10 000 approximate permutation distribution by randomly exchanging pairs of Y elements (lmPerm package for R) was performed. All results are presented as estimated mean ± standard error. The degrees of freedom and F-scores were reported and varied between permutation and classical tests.

## 3 Results

### 3.1 Ground reaction force analyses

As illustrated on Figure 1 (B & C), each lower limb produced a major normal force component in the direction of the sled displacement (performance axis) as well as a downward reaction force along the antero-posterior axis and lateral forces in opposite directions, leading to a resultant lateral force close to zero along the medio-lateral axis (Figure 1A). Regarding each ground reaction force variable, significant effects were found for sex, load and for recovery session. The results are shown in Table 1.

**Table 1:** Summary of statistical effects for the ground reaction force variables.

		Sex	Load	Session	Sex × Load	Sex × Session
Bilateral	Normal force	∗∗∗	∗∗∗	NS	∗	∗
	Antero-posterior force	NS	∗∗∗	∗	NS	NS
	Medio-lateral force	NS	NS	NS	NS	∗∗
	Effective force ratio	∗∗	∗∗	∗	NS	NS
	Rate of force development	∗	NS	NS	NS	NS
Dominant lower limb	Normal force	∗∗∗	∗∗∗	NS	∗	∗
	Antero-posterior force	NS	∗∗∗	∗	NS	NS
	Medio-lateral force	NS	∗∗∗	NS	NS	NS
	Effective force ratio	∗∗	∗∗	NS	NS	∗
	Rate of force development	∗	NS	NS	NS	NS
Non-dominant lower limb	Normal force	∗∗∗	∗∗∗	NS	∗	NS
	Antero-posterior force	NS	∗∗∗	∗	NS	NS
	Medio-lateral force	NS	∗∗∗	NS	NS	NS
	Effective force ratio	NS	NS	NS	NS	NS
	Rate of force development	∗∗	NS	NS	NS	NS

Statistical difference: ∗p < 0.05, ∗∗p < 0.01, ∗∗∗p < 0.001; NS: non-significant.

ANOVA by permutation of the bilateral mean force production revealed load- (F_(1,345)_ = 304.8) and sex- (F_(1,15)_ = 35.4) dependent effects (p < 0.001), and interaction (p < 0.05) on the normal force component: All resistive loads, except 40% from 20% BW (p = 0.069), differed from each other (106 ± 13 N; p < 0.001), and women produced lower mean force values than men (−310 ± 49 N; p < 0.001), but this difference decreased along recovery (−303 ± 48 N at PRE; -290 ± 45 N at D2 and -273 ± 47 N at D4). Analysis of the antero-posterior force component showed a load effect (F_(3,357)_ = 176.8, p < 0.001), with greater values at 60% BW (49 ± 8 N; p < 0.001), and a session effect with lower values at D4 compared to PRE (−39 ± 13 N; p < 0.01). Analysis of the medio-lateral force component showed a sex × session interaction (F_(2,17)_ = 7.6, p < 0.01), with higher force values at D4 compared to D2 for men only (10 ± 13 N; p < 0.01). Analysis of the dominant *vs.* non-dominant lower limb revealed similar results except for the normal force component, which showed no sex × session interaction for the non-dominant limb. For the medio-lateral force component, only a load effect was found for each lower limb (F_(3,345)_ = 25.2 and F_(3,357)_ = 53.9 for dominant and non-dominant limb respectively, p < 0.001): The medio-lateral force at 0% BW was lower than at 40% and 60% BW for the two lower limbs (p < 0.01) and lower than at 20% BW for the sole dominant-limb (p < 0.05).

In all testing conditions, women showed a lower effective force ratio than men for the dominant limb (−5.5 ± 1.8%; p < 0.01) and bilaterally (−4.5 ± 1.6%; p < 0.01). Independently of sex, the bilateral ratio was slightly increased at D4 compared with PRE (2.1 ± 0.8%; p < 0.05). Only the dominant limb showed a sex × session interaction (F_(2,345)_ = 3.4, p < 0.05) because of a smaller difference between women and men at D4 (−4.6 ± 1.6%) than at PRE (−5.6 ± 1.8%) and D2 (−5.7 ± 1.9). Load factor also influenced the effective force ratio (F_(3,345)_ = 4, p < 0.01), showing a lower value at 60% than at 20% BW (−1.3 ± 0.5%; p < 0.05) except for the non-dominant limb. A main effect of sex was found for the rate of force development (F_(1,15)_ = 6.8, p < 0.05), which was lower in women than in men (−3915 ± 1406 N/s; p < 0.01) independently of the load and session.

### 3.2 Force-velocity relationship

The FV slope based on automatically selected trials was 82.4% similar to the slope based on manual selection. The number of trials selected averaged 7.2 ± 1.2 (maximum was 8 points) regardless of sex and session, with a similar R^2^ (0.80 ± 0.13). Significant effects of sex, load, and recovery session were found for most variables (Table 2).

**Table 2:** Summary of statistical effects for the FV and EMG variables.

			Sex	Load	Session	Sex × Load	Sex × Session
Force Velocity parameters	Bilateral	$\bar{\text{F}}$ 0	∗∗		NS		NS
		$\bar{\text{V}}$ 0	∗∗∗		NS		∗
		$\bar{\text{P}}$ max	∗∗∗		NS		∗
		S_FV_	NS		NS		NS
		SI_SFV_	NS		NS		NS
	Dominant lower limb	$\bar{\text{F}}$ 0	∗∗		NS		NS
		$\bar{\text{V}}$ 0	∗∗∗		NS		∗
		$\bar{\text{P}}$ max	∗∗∗		NS		∗
		S_FV_	NS		NS		NS
		SI_SFV_	NS		NS		NS
	Non-dominant lower limb	$\bar{\text{F}}$ 0	∗∗		NS		NS
		$\bar{\text{V}}$ 0	∗∗∗		NS		∗
		$\bar{\text{P}}$ max	∗∗∗		NS		∗
		S_FV_	NS		NS		NS
		SI_SFV_	NS		NS		NS
Mean muscle activity	RMS global push-off		∗∗∗	NS	∗∗∗	NS	∗∗∗
	RMS first part of the push-off		∗∗∗	∗∗∗	∗∗∗	NS	∗
	RMS middle part of the push-off		∗∗∗	NS	∗∗∗	NS	∗∗∗
	RMS last part of the push-off		∗∗∗	NS	∗∗	NS	∗∗
Muscle synergies	Temporal synergies	CoA Motor primitive 1	∗∗∗	NS	∗	∗	∗∗∗
		FWHM Motor primitive 1	NS	NS	NS	NS	NS
		CoA Motor primitive 2	NS	NS	NS	∗	∗∗∗
		FWHM Motor primitive 2	NS	NS	NS	NS	NS
		CoA Motor primitive 3	NS	NS	NS	NS	NS
		FWHM Motor primitive 3	NS	NS	NS	NS	NS
	Spatial synergies	Motor modules synergy 1	∗∗∗	NS	NS	NS	NS
		Motor modules synergy 2	∗	NS	NS	NS	NS
		Motor modules synergy 3	∗	NS	NS	NS	NS

Statistical difference: ∗p < 0.05, ∗∗p < 0.01, ∗∗∗p < 0.001; NS: non-significant. Grey boxes are used when the effect is inappropriate. For clarification reasons, muscle and trial factors are not shown. For the synergy analysis, only post-hoc of the interactions are shown. NS: non-significant (p > 0.05).

**Figure 4:**
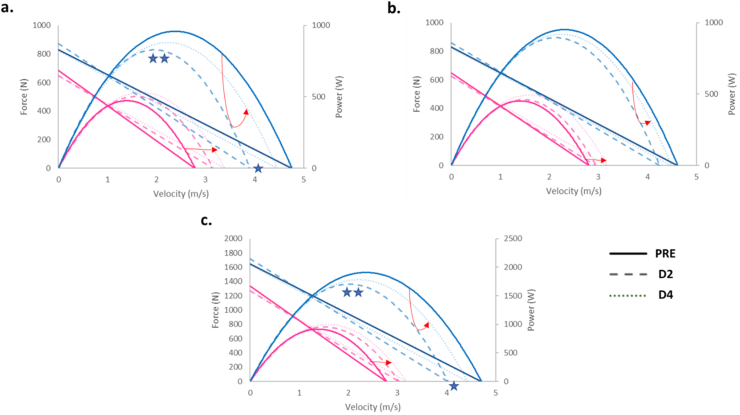
Group mean Force-velocity and velocity-power relationships for dominant (a), non-dominant (b) and both (c) lower limbs between men (blue curves) and women (pink curves) as function of session: PRE, D2 and D4 in bold, light and very light respectively. The red arrows indicate the session order. ∗: p < 0.05 and ∗∗: p < 0.01 compared to PRE for men. Sex-differences were statistically significant (p < 0.001) in $\bar{F}$ 0, $\bar{V}$ 0 and $\bar{P}$ max.

As shown in Figure 4, women showed lower values of $\bar{\text{F}}$ 0 (F_(1,15)_ = 12.6, p < 0.01), $\bar{\text{V}}$ 0 (F_(1,17)_ = 39.1, p < 0.001), and $\bar{\text{P}}$ max (F_(1,17)_ = 122.3, p < 0.001) than men. $\bar{\text{V}}$ 0 and $\bar{\text{P}}$ max showed a sex × session interaction (F_(2,34)_ = 4.7 and F_(2,34)_ = 4.8 respectively, p < 0.05): Only men showed lower $\bar{\text{V}}$ 0 and $\bar{\text{P}}$ max at D2 compared to PRE, for both lower limbs (−0.68 ± 0.23 m/s, p < 0.05 and -205 ± 66 W, p < 0.01, respectively) (Figure 4C) and for the dominant limb (−0.85 ± 0.28 m/s, p < 0.05 and -130 ± 36 W, p < 0.01, respectively) (Figure 4A). No significant effects were found for the slope and asymmetry index parameters (S_FV_ and SI_SFV_).

### 3.3 Mean muscle activity

**Figure 5:**
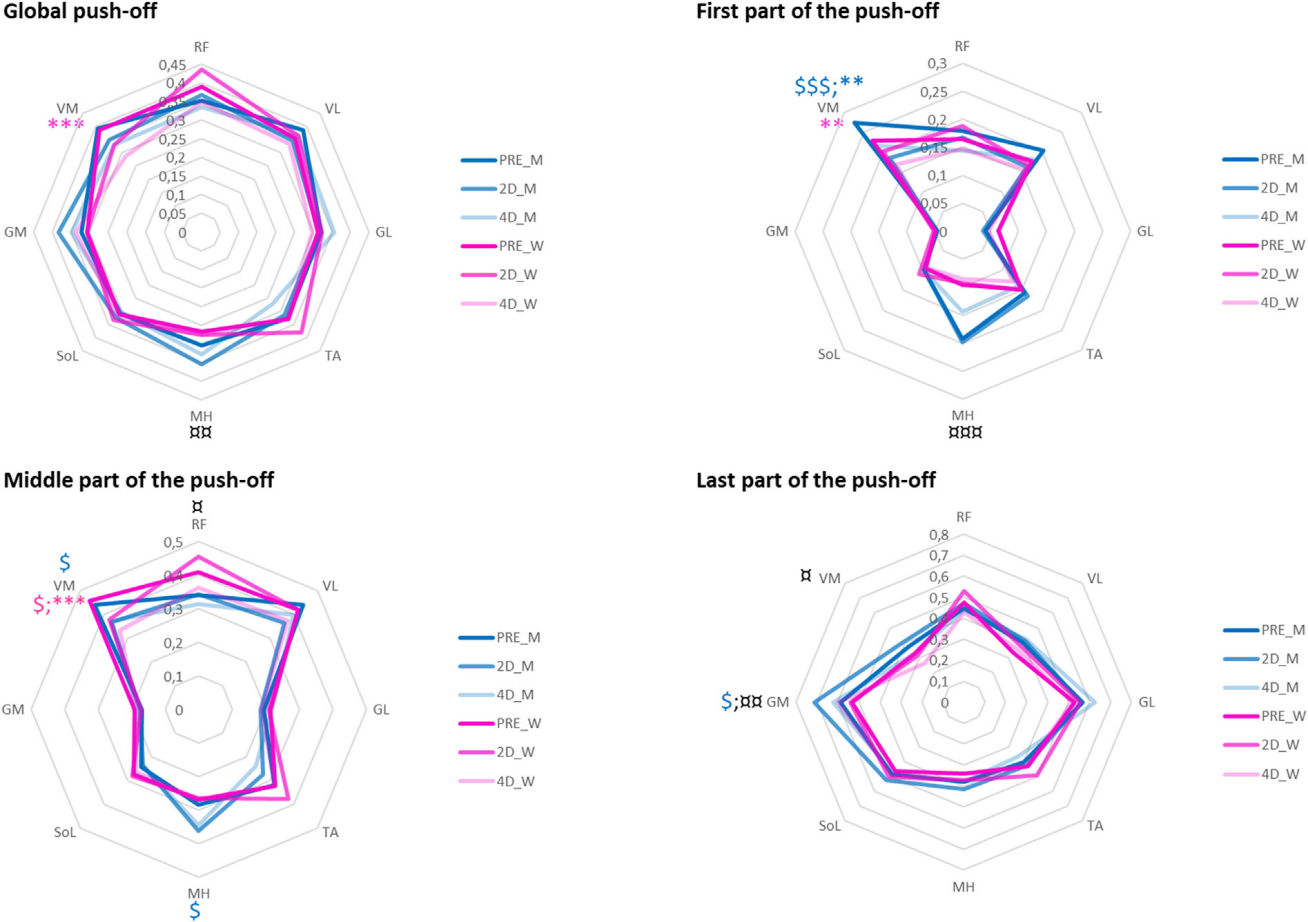
RMS of normalized muscle activation values averaged for women (pink curves) and men (blue curves) for each session: PRE, D2 and D4 in bold, light and very light respectively; and each part (global, middle, first and last part) of the push-off. Muscle abbreviations: RF, rectus femoris; VM, vastus medialis; VL, vastus lateralis; MH, medial hamstrings; TA, tibialis anterior; GM, gastrocnemius medialis; GL, gastrocnemius lateralis; SO, soleus. “$” and “∗” represent significant differences at D2 and D4 as compared to PRE, respectively. “¤” indicates a significant sex difference. The number of symbols indicates the statistical level: one for P < 0.05, two for p < 0.01 and three for p < 0.001 in pink for women and in blue for men.

The statistical analysis of muscle EMG-activity revealed significant effects of sex, load, and recovery session on the RMS values. The relative muscle activity changes (expressed in percentage of the maximal values at PRE) were dependent on the push-off phase considered (i.e. global, first, mid-, or last part of the push-off) (Table 2 and Figure 5).

For the global push-off (Figure 5A): Vasti muscles showed the greatest average relative activity during the push-off (p < 0.01). Considering the entire group of participants, vastus medialis activity decreased at both D2 and D4 compared with PRE (−5 ± 1.6 %, p < 0.05 and -8.2 ± 1.6%, p < 0.001, respectively). Women showed lower relative medial hamstring activity than men (5.6 ± 1.7%, p < 0.01) independently of the session, and reduced vastus medialis activity (−10 ± 2.3%, p < 0.001) at D4.

For the first part of the push-off (Figure 5B): Vastus medialis muscle showed the highest relative activity (21 ± 0.9%) compared to other muscles and greater relative rectus femoris activity was found at 60% BW than at other loads (−5.5 ± 1.1%, p < 0.01). Vastus medialis activity was reduced at D2 and D4 for the whole group (D2: -5.8 ± 1.1% and D4: -5.9 ± 1%, p < 0.001) and for men (D2: -8.9 ± 1.6%, p < 0.001; D4: -5.9 ± 1.5%, p < 0.01) but at D4 only for women (−6 ± 1.4 %, p < 0.01). Women also showed lower relative medial hamstring activity than men at PRE (−9.6 ± 2.2%, p < 0.001) and D2 (−10.4 ± 2.5%, p < 0.001), but not at D4 (p = 0.067).

For the mid-part (Figure 5C): Vasti muscles had the highest relative activity (39.7 ± 1.2%) compared to other muscles. Independently of session, women had higher relative activity of rectus femoris (7.6 ± 2.3%, p < 0.01) and tibialis anterior (6.3 ± 2.3%, p < 0.05), but lower relative medial hamstring activity (6.3 ± 2.3%, p < 0.05) than men. Vastus medialis activity was reduced at both D2 and D4 for the whole group (D2: 7.7 ± 1.7% and D4: 9.8 ± 1.8%, respectively, p < 0.001) and for women (D2: −8.2 ± 2.3%, p < 0.05 and D4: −12.5 ± 2.5%, p < 0.001). Men showed at D2 a decreased vastus medialis activity (−7.8 ± 2.4%, p < 0.05) and increased medial hamstring activities (7.9 ± 2.4%, p < 0.05).

For the last part (Figure 5D): Both gastrocnemii muscles were highly activated (57.9 ± 1.2%). Women showed lower relative gastrocnemius medialis (−8.2 ± 2.5%, p < 0.01) and vastus medialis (−6.7 ± 2.5%, p < 0.05) activities than men. At D2, men showed increased gastrocnemius medialis activity (12.5 ± 3.5%, p < 0.05) that resulted in an increased sex difference (17 ± 4.3%, p < 0.01).

### 3.4 Muscle synergies

**Figure 6:**
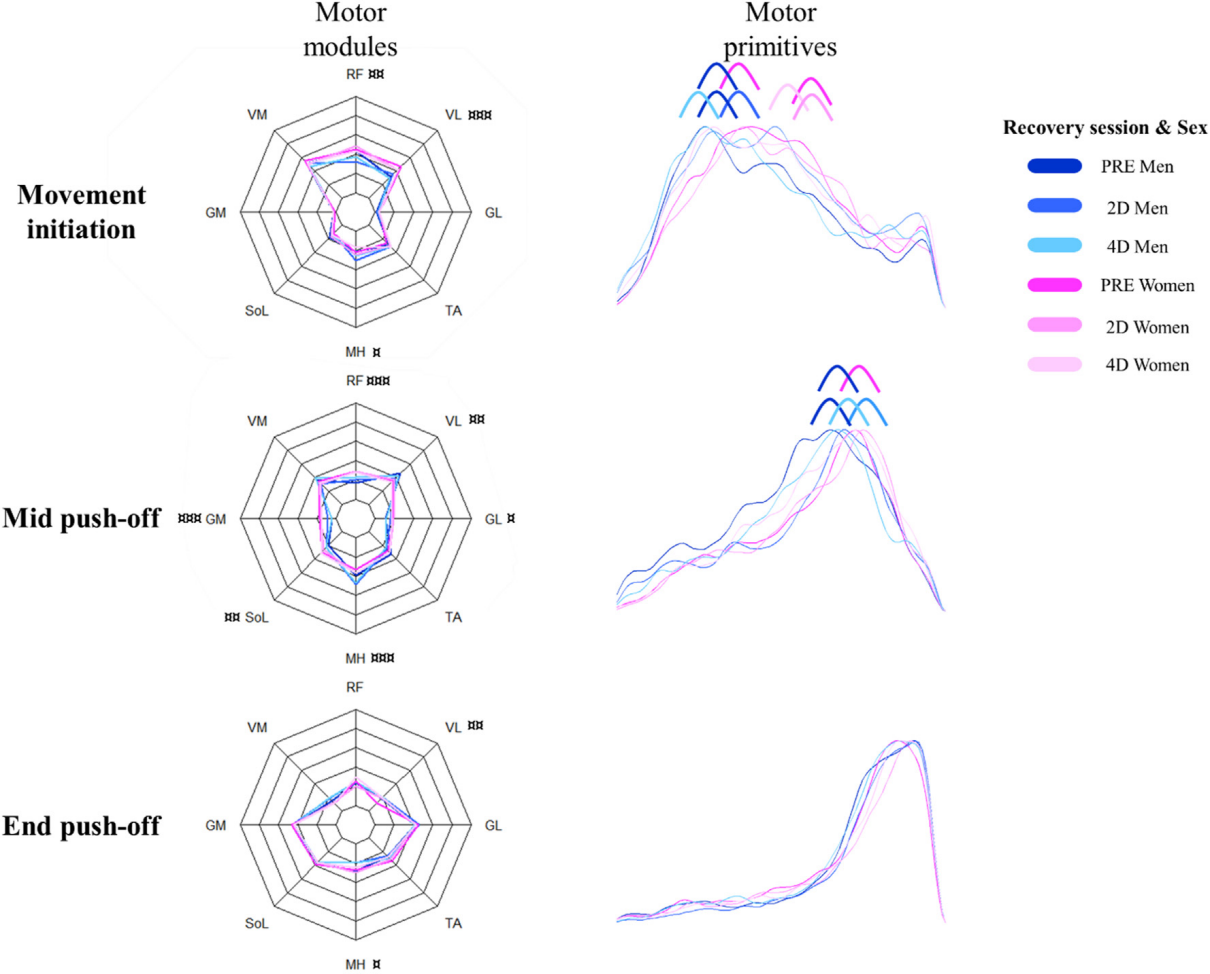
Motor modules and motor primitives (averaged for each participant and load excepted 20% BW condition) of the three fundamental synergies for each recovery session (PRE, D2 and D4) in women and men. The motor modules are presented on a normalized y-axis base: each muscle contribution within one synergy can range from 0 to 1. For the mean motor primitives, the x-axis full scale represents the normalized push-off and the y-axis the normalized amplitude. Muscle abbreviations: RF, rectus femoris; VM, vastus medialis; VL, vastus lateralis; MH: Medial Hamstrings; TA, tibialis anterior; GM, gastrocnemius medialis; GL, gastrocnemius lateralis; SO, soleus. “¤” indicates a significant sex difference. The number of symbols indicates the statistical level: one for P < 0.05, two for p < 0.01 and three for p < 0.001. Difference of center of activity of the motor primitive between men and women is indicated by blue and pink curvy symbols, and inter-session differences by curvy symbols of similar colors (blue or pink).

The EMG activity recorded from 8 muscles of the lower limb could be factorized into three task-related muscle synergies in all test conditions (Figure 6). A good reconstruction quality was obtained by the NMF for all conditions (for men: R^2^ = 0.910 ± 0.002 and for women: R^2^ = 0.908 ± 0.002), with negligible effect of load (p < 0.001): The R^2^ was slightly higher at 0% and 20% BW than at 60% BW (0.025 ± 0.009; 0.026 ± 0.009 respectively, p < 0.05).

The three fundamental synergies occurred at specific phases of the push-off: the first synergy at the push initiation with a major influence of knee extensors (p < 0.001). The second synergy at the mid push-off, considered as the transfer between the other 2 synergies, showed an overall muscle contribution especially in women. The third synergy at the end of the push-off phase, showed a primary contribution from the ankle extensors. The mid push-off was not found at the 20% BW condition in the PRE-session where both sexes exhibited only the movement initiation and the end push-off synergies (Figure 6). Thus, the 20% BW condition was not included in the linear mixed model.

For each muscle synergy variable (i.e., CoA, FWHM and motor modules; Table 2), the main significant results are summarized in the following section, first independently of the recovery session and then as a function of it:

At the push initiation (first synergy), women had higher rectus femoris (0.07 ± 0.02, p < 0.01) and vastus lateralis (0.11 ± 0.02, p < 0.001) contributions, but lower medial hamstring contributions (−0.05 ± 0.02, p < 0.05) than men. For both sexes, vastus medialis was the most activated muscle (0.50 ± 0.02 for men and 0.52 ± 0.02 for women, p < 0.001). The center of activity (CoA) of the motor primitive was located at 41.6 ± 0.6% of the push-off time, but was reached later by women at 40% BW (4.2 ± 1.5%, p < 0.05) and 60% BW (9.7 ± 1.5%, p < 0.001). The duration of the main activity of motor primitive (quantified by the FWHM parameter) did not differ depending on either sex or load condition although it tended to be longer in women than in men (p = 0.07).

Regarding the mid-push off (second synergy) in men, medial hamstring and vastus lateralis were the most activated muscles (0.45 ± 0.02; 0.37 ± 0.02, respectively, p < 0.05). Compared to men, women presented relatively smaller contributions from the medial hamstring (p < 0.001) and vastus lateralis (p < 0.01), but greater contributions from the triceps surae (gastrocnemii and soleus) and rectus femoris (p < 0.05). The CoA was located at 63.2 ± 0.5% of the push-off time and was reached later by women at 60% BW (5.9 ± 1.5%, p < 0.001) compared to men. Neither resistance conditions nor sex differed in the duration of the main activity of motor primitive.

At the end of the push-off phase (third synergy), and in contrast to the sex differences in the other synergies, women had greater contributions of medial hamstring (0.05 ± 0.02, p < 0.05) and lower vastus lateralis (−0.06 ± 0.02, p < 0.01) muscles than men. The CoA was located at 80.7 ± 0.3% of the push-off time. Independently of sex, the main activity of this motor primitive was shorter in duration (FWHM) than that of the first (−13.9 ± 2.4, p < 0.001) and second (−10.7 ± 2.4, p < 0.001) motor primitives.

The recovery session did not influence motor modules and FWHM, but impacted CoA as follows: A synergy × sex × recovery session interaction was found (p < 0.05). For the first synergy, men showed a CoA reached later at D2 than at PRE (4.1 ± 1.4%, p < 0.01). At D4, both men and women showed a CoA reached earlier than at PRE (−3.7 ± 1.4%, p < 0.05 and -4.8 ± 1.4%, p < 0.01, respectively) and D2 (−7.9 ± 1.5%, p < 0.001 and -6.0 ± 1.4%, p < 0.001, respectively). Thus, women showed a delayed CoA compared with men at PRE and D4, but not at D2. For the second synergy, men showed: Delayed CoA at D2 (6.5 ± 1.5%, p < 0.001) and D4 (3.1 ± 1.5%, p < 0.05) compared with PRE, and earlier CoA at D4 than at D2 (−3.4 ± 1.5%, p < 0.05). The 20% BW was characterized for both sexes by only 2 synergies at PRE *vs*. 3 synergies at D2 and D4. The PRE-session lacked the second synergy corresponding to the mid push-off, while showing a longer first synergy and an unchanged third synergy (Figure 6).

## 4 Discussion

This study was designed to explore the influence of sex on the FV profile and neuromuscular pattern when performing a ballistic multi-joint HFV test before a graded running race and at 2 and 4 days of its delayed recovery phase. Independently of the load and session, women produced lower mean force values, showed a lower effective force ratio and a lower rate of force development than men, that resulted in lower $\bar{\text{F}}$ 0, $\bar{\text{V}}$ 0 and $\bar{\text{P}}$ max values. Supporting partially our first hypothesis of a lesser change in the FV profile in women than in men during the delayed recovery phase, only men showed significant decrements in $\bar{\text{V}}$ 0 and $\bar{\text{P}}$ max but not in $\bar{\text{F}}$ 0. On the other hand, confirming our second hypothesis, the muscle activity pattern was found as sex-dependent before fatigue, with sex-specific changes along the 4 days of recovery.

Confirming the interest of using the FV profile for assessing sex influence on the functional fatigue effects, the FV relationship presented a good linearity (measured by R^2^) in all recovery sessions and revealed significant sex-dependent fatigue effects that were not detected by the classical analyses using the global mean force value [18]. The discrepancy between the two analyses could be attributed to the fact that the FV relationship included most of the performed push-off trials whereas the classical analysis was limited to the best trial per resistive load. As the inter-trial variability may be expected to change with fatigue [51], the FV profile is considered as pertinent as it includes most of the trials. Extrapolation of the extreme force ($\bar{\text{F}}$ 0), velocity ($\bar{\text{V}}$ 0) and power ($\bar{\text{P}}$ max) values may also be considered relevant to reveal small changes in this purely concentric multi-joint task known to favor inter-muscular compensations leading to no fatigue-induced drop in performance [10]. Vuorimaa et al. (2006) [24] even found a performance improvement in a loaded half-squat test after running until exhaustion.

In the present experiment, only men showed decreases of $\bar{\text{V}}$ 0 and $\bar{\text{P}}$ max at D2 when averaged for both limbs and also for the dominant limb alone. As expected, these functional decrements were limited compared to those found at D2 and D4 for these male runners in more stressful drop jump (jump with ground impact) and bilateral MVC (monoarticular knee extension) tests [18]. The lack of delayed functional deficits found for the present female endurance runners is consistent with their reported lower sensitivity to muscle damage [52, 53] and shorter inflammatory process [54, 55]. Among the potential underlying mechanisms, women are considered to have a proportionally larger area of slow twitch muscle fibers (known as more resistant to metabolic fatigue and mechanical stress) and to benefit from the direct and indirect effects of estrogen (on muscle membranes, perfusion and metabolism) [11, 12, 13, 20].

On the other hand, the unexpected lack of change in $\bar{\text{F}}$ 0 during the delayed recovery period suggests that the 20-km graded running race impacted more the velocity than the force capacity. These findings differ from the greater decrease in $\bar{\text{F}}$ 0 than $\bar{\text{V}}$ 0 reported in male runners at the end of longer races (60 and 100 km UTMB races) with larger gradient [56] than the present one. Any comparison with the present study is unfortunately limited by the obvious differences in running distance, gradient, testing time (acute *vs.* delayed) and testing task (cycling *vs*. ballistic jump). The decrease in $\bar{\text{V}}$ 0 means a decrease in the ability of runners to produce vertical force at high speeds, which could increase the risk of injury involving a negative influence on the quality of specific sport skills [57]. The decrease of $\bar{\text{V}}$ 0 and $\bar{\text{P}}$ max in men may be favored on this particular ergometer since the push-off starts at low pre-activation level and higher extrapolated values of $\bar{\text{V}}$ 0 (4.7 m/s in PRE for men) are obtained compared to the 2–4 m/s generally reported when using the classical vertical squat jump [58]. Finally, the unilateral fatigue effects found at D2 may be attributed to the asymmetry reported for the knee extensor strength, running kinetics and kinematics of endurance runners [27]. For the present runners, confounding factors may be considered such as their recreational running level, the positive and negative gradients of the 20-km race and their limited participation to this graded race. Furthermore, the unilateral fatigue effects revealed by the bilateral HFV test confirms the lack of “cross-over effects” previously reported in a maximal bilateral jump test performed D2 after exhaustive unilateral rebounds [59]. Maximal bilateral jump test may thus be used to examine fatigue effects in each lower-limb separately.

Supporting our second hypothesis, women showed a different EMG pattern than men in the HFV test, and different neural adjustments at D2 and D4. The respective analyses of mean muscle activity and muscle synergies agreed with each other and revealed complementary findings. Independently of the recovery sessions, men and women clearly differed regarding the solicitation of the bi-articular thigh muscles (medial hamstring and rectus femoris). Compared to men, women showed lower activity and relative contribution of the medial hamstring muscles during the global push-off, especially at the push initiation and during the subsequent mid-phase. Considering the lower peak torque and velocity of the hamstring/quadriceps ratio reported for women [60, 61], the contribution of the medial hamstring muscles to the horizontal push-off may be expected to be lower in women than in men. Conversely, women presented a higher relative contribution of rectus femoris to the initiation and mid-push phases. Although men and women initiated the push from the same hip and knee joint angles (set at 90°), the greater reliance of women on the rectus femoris rather than on the hamstring muscle group as men could be considered a disadvantage due to the non-optimized muscle length of the rectus femoris [62]. At the push-initiation, women thus relied more on the knee than on the hip extensors whereas men relied much on both. Analysis of the synergies revealed sex-specific characteristics in both spatial and temporal structure of the muscle synergies, notably for the second synergy, which showed that men activated selectively their vasti and medial hamstring muscle groups whereas women involved also the rectus femoris and the triceps surae (soleus and gastrocnemii). This global involvement of the lower limb muscles in women seems to have resulted in some loss of energy given their lower effective strength ratio. Furthermore, the first and second motor primitives exhibited a later center of muscle activity in women than in men, which may have contributed to the lower rate of force development in women.

Regarding the neural adjustments to fatigue, the RMS analysis of the dominant lower limb revealed for both men and women a reduced vastus medialis activity at D2 and D4 (especially in the mid-phase of the push). This result is in agreement with the delayed decreases found for these runners in the knee extension MVC tests [18]. In the present HFV test, although men showed at D2 increased activities of the medial hamstrings at mid-push and of the gastrocnemius medialis in the final push-off phase, these compensations were not sufficient to compensate for the loss of vastus medialis activity as shown by the reduced $\bar{\text{V}}$ 0 and $\bar{\text{P}}$ max. The short duration of the end push-off synergy (where the contribution of the triceps surae was the most important) may also have limited the potential compensatory role of this muscle group. Interestingly also, as women relied on a more evenly distributed involvement of all lower limb muscles than men at mid-push-off, the fatigue-induced decrease in vastus medialis activity is likely to have had a lesser functional impact on their performance. On the other hand, although women reported specific DOMS from the hamstrings [18], the fact that they activated less than men this muscle group in the HFV test is likely to have limited its negative influence.

While no change was found with respect to the motor modules of each synergy, men exhibited a delayed center of activity (CoA) at D2 in the first and second motor primitives, so that their earlier CoA than women at PRE no longer differed at D2. At D4, the medial hamstring activity did not differ anymore between men and women at the push initiation and both sex groups showed earlier CoA than at PRE for the first motor primitive, and for the second motor primitive also for men only. Interestingly, these temporal shifts of CoA paralleled the functional changes: Men showed delayed CoA and decreased $\bar{\text{V}}$ 0 and $\bar{\text{P}}$ max at D2, followed by earlier CoA and a return to their PRE-functional values at D4. Women showed earlier CoA, improved effective force ratio, and lesser functional sex difference at D4 suggesting a potential learning effect of the ballistic task. Finally, the 20% BW condition differed from the others by showing for both sexes only two synergies at PRE, but three synergies at both D2 and D4 that could reflect a fatigue effect for both sexes. However, as about 30% of combined synergies were found for the 20% BW at PRE for both men and women compared to about 15% for the other loads, this result may still be attributed to the variability of the computation method [30].

Some methodological limitations need to be addressed. First, the small sample size in this study is a major limitation as we did not have the number of participants required for 80% statistical power. Thus, we calculated a second time (a posteriori) the statistical power based on the theoretical maximal power ($\bar{\text{P}}$ max) indicating that the statistical power was of about 45% regarding the interaction effect. It is important to note that sex-fatigue differences in terms of functional parameters were only found for pure theoretical values of the FV profile. Differing from the vertical squat jump test, the present ballistic ergometer was particular since the squat jumps were performed while lying in a supine position with little pre-activation compared to a vertical push. Using this ergometer, higher extrapolated values of $\bar{\text{V}}$ 0 were obtained compared to those using the classical vertical squat jump [58]. In this context, Lindberg et al. (2021) [63] reported high repeatability for all FV parameters on a leg press (close to our ergometer) that was attributed to the highly standardized position [63]. Although the present FV was calculated for each lower limb separately, which is interesting as they were impacted differently by fatigue, it should be mentioned that the same mean velocity was applied for both lower limbs. Based on the asymmetrical fatigue effects, it could be interesting to perform also unilateral tests. Concerning the EMG analysis, the model of muscle synergies must be considered as perfectible since only 8 muscles of one lower-limb were included so that the gluteus maximus, expected to play a major role at the initiation of the push-off, was not recorded.

## 5 Conclusion

This study provides new insights into the impact of graded endurance running on functional and neuromuscular recovery between men and women. First, women were found to recover earlier in a dynamic multijoint push-off task as only men showed decreases in $\;\bar{\text{V}}$ 0 and $\bar{\text{P}}$ max at D2. This ballistic force-velocity test revealed clear sex-differences in the lower limb activities and muscle synergies both before fatigue and during the recovery phase suggesting that the coordination analysis may be of interest to reveal neuromuscular fatigue and different neural strategies. Interestingly, although both men and women showed reduced activity of the vastus medialis muscle up to D4, only male runners showed a delayed timing of the center of activity of the muscle synergies at D2 before returning to baseline values at D4. The lesser influence of this fatigued quadriceps muscle on the women synergies is attributed to their use of a more evenly distributed activation of lower limb muscles than men. Despite the earlier functional recovery of women, additional results are needed to define their optimal time to resume running. In future studies, it would be of interest to repeat such analyses in running to better appreciate the richness of the intermuscular compensations of men and women with fatigue.

## Declarations

### Author contribution statement

Author name as it appears on the manuscript: Conceived and designed the experiments; Performed the experiments; Analyzed and interpreted the data; Contributed reagents, materials, analysis tools or data; Wrote the paper.

Robin Macchi & Caroline Nicol: Conceived and designed the experiments; Performed the experiments; Analyzed and interpreted the data; Wrote the paper.

Arnaud Hays: Conceived and designed the experiments; Performed the experiments; Analyzed and interpreted the data.

Fabrice Vercruyssen: Conceived and designed the experiments; Performed the experiments.

Alessandro Santuz, Avner Bar-Hen & Adamantios Arampatzis: Analyzed and interpreted the data.

### Funding statement

Robin Macchi was supported by the GDR Sport & Activité Physique.

### Data availability statement

Data will be made available on request.

### Declaration of interests statement

The authors declare no conflict of interest.

### Additional information

No additional information is available for this paper.
